# On the Use of Mercury in Syphilis

**Published:** 1831

**Authors:** James M. Staughton

**Affiliations:** Professor of Surgery in Miami University


**Art. III.** *On the use of Mercury in Syphilis.*—By James M. Staughton, *Professor of Surgery in the Miami University. Read before the Cincinnati Medical Society.*

The tenacity with which the human mind clings to its earliest impressions, is one of its most interesting properties. Though it sometimes impedes us in the search of truth, it more frequently prevents the hasty adoption of error, and forcibly urges that philosophic doubt, which marks the progress of every accurate mind, in the investigations of science.

Most of us in the first rudiments of our medical knowledge, were taught to believe, that in Syphilis, mercury was the solum et unicum remedium; we were taught to regard it as the sheet anchor, as the ultima ratio.—Did we not step forward into practice with the firm conviction, that in all cases of the venereal disease we had a sovereign balm? Did we not look at those unfortunate cases, in which the disease was aggravated by mercurials, as the results of malpractice, and have we not regarded our professional brethren under whose treatment such cases have occurred, with doubts and suspicions as to their faith in the remedy, and the judgment with which they have employed it? I fear we have—I, at any rate, am free to confess that I have. I do not recollect any conviction that was so deeply rooted in my mind as that of the infallibly specific powers of mercury in Syphilis. But twelve years have taught me that there *are* some forms of venereal disease, that not only refuse to yield to the powers of mercury, but which are increased in violence, by its exhibition.—My early prejudices are now, in a manner, overcome, for I have discovered that some of them were founded in error.

It is not my intention to lay before this society a confession of my faith, but to take a rapid glance at the changes in the professional opinions of this subject, that have been wrought in the United States within the space of a few years. I am aware, that the usual limits of a paper will not allow me to do justice to the subject, and can scarcely expect to throw any light upon it, but I shall be gratified if I shall succeed in presenting matter for discussion, and if I shall be able to elicit from the senior members of the society an expression of their views, sanctioned by extended observation and experience.

The very valuable work of John Hunter on Syphilis established the use of mercury. It cannot be denied, however, that previous to his time, surgeons were found in various parts of the world, who made a practice of curing this disease by other means. It was understood, too, and fairly admitted, that in warm climates the venereal disease lost much of its severity, and was frequently cured by diaphoretic, and other mild measures. But Hunter's book came out with a weight of authority almost irresistable, and boldly placed in an imposing attitude, that the primary ulcer of Syphilis could not be cured without mercury, but would go on, becoming progressively worse and worse. The doctrine was at once adopted in Europe and brought over to this country by his pupils, who promulgated it here with that ardor and enthusiasm that has characterized all their efforts. With the first teaching of medical science in the United States, therefore, was the specific virtue of mercury enforced.

Several of the constitutional affections which were noticed in the mercurial treatment, such as the ulcers in the throat, blotches on the skin, nodes on the bones, and pains in the limbs were, by some, attributed to the employment of the mercury. Considerable excitement prevailed on this subject; the friends of the established method insisting, that it was the natural progress of the disease, kept under indeed by sanative powers of the remedy, while the heterodox were loud in the abuses which they heaped on the mercury. This seemed to open the eyes of medical men, and immediately close and accurate observations were instituted. It was soon perceived that there were cases of venereal ulcers, which, even though salivation had been produced, would run on to constitutional lues, and that under these circumstances, mercury most certainly and absolutely aggravated it.—With great slowness and much caution was this fact admitted, but admitted it was, for I believe the most strenuous advocate of the malpractice would not dare to deny its correctness. Mercury then was considered as injurious in some of the ulcerations of the penis, which eventuate in constitutional symptoms.

While these subjects were held by the profession under consideration, intelligence was brought from Europe of new and imposing views of Syphilis. Surgery unlike other branches of science is not retarded in its progress by war. During the numerous and varied conflicts that succeeded the French Revolution, many important additions were made to the stock of chirurgical knowledge.—Men, collected in immense masses, afforded ample field for the observations of those learned and intelligent surgeons, that were placed high in command in the respective armies.

The British surgeons, observing the mildness which the venereal diseases wore on the sunny plains of the Peninsula, were induced to try the effects of other then mercurial remedies.—They quickly perceived that cleanliness, a few saline cathartics and the use of antimonials were all that was necessary, in addition to copious dilutions of guaicum and sarsaparilla, to cure most of the cases of chancre that presented.

The next step was perfectly natural. They introduced the same mode of treatment into the British Islands and found similar success to attend it.

The immense number of cases which have been reported, by the army board, of cases treated in England, Ireland and Scotland on these principles has given the practice a high and commanding reputation.

Without going more extensively into the historical details; I shall take up the examination of the following questions as being perfectly relevant to the present state of our knowledge on this subject.

1. Does the use of mercury form or contribute to form any of the constitutional symptoms of Syphilis?

2. Does mercury ever aggravate any form of Syphilis?

3. Are there any forms of Syphilis that can be cured without mercury?

4. Is there reason to believe that there is more than one form of venereal virus?

5. Is there reason to believe that the venereal poison has undergone any change in its nature or virulence.

6. Is mercury *ever* necessary in the cure of Syphilis?

To the first question, does mercury form or contribute to form any of the constant symptoms of Syphilis? I return boldly and unhesitatingly, the answer, no! The reaction which has occurred in the professional sentiments of some, as to the value of the remedy, is as violent against it, as were the previous prejudices blind and obstinate. Every possible variety of injury is now ascribed to the use of mercury. We want proof, however, of the *existence* of these injuries. For, in the first place, it is known, that the usual train of constitutional symptoms, will arise in a neglected case of true chancre, where not one single atom of mercury has been given.

In the second, we know that mercury, when administered in large quantities for the cure of other complaints, does not produce these symptoms. The testimony of Mr. Carmichael, on this point, is, by far, too important to be omitted. "I beg leave to observe, that I have not, nor do I believe that any person has, witnessed ulcers on the skin, and throat, and nodes, or the bones, from the exhibition of the most extensive courses of mercury, in any other than venereal diseases, nor even an eruption, except the well known mercurial eczema."

The establishing of these two points, in my opinion, settles the question most absolutely. We pass on, therefore, to the second question. Does mercury ever aggravate any venereal symptoms? I shall never forget the manner in which my mind was brought to the conclusion, that it was possible for mercury to act in this way. A gentleman applied to me with a large ulcer on the back part of his throat, covered by coagulable lymph, which resembled a piece of thick wash leather, glued on. I pronounced it, at once, a venereal affection, and confidently promised him a speedy and effectual cure. I employed mercurials, in the firm expectation, that so soon as his system came to feel the mercurial impression, the ulcer in the throat would experience a decided improvement. I was much surprised, however, to find, that as the action of the remedy increased, the throat became worse, the original ulcer spread, another formed, and before I was well aware, a considerable portion of the soft palate was implicated in the ulcerative action. Very fortunately for the patient, about this time, he lost all confidence in me and my remedy, and withdrew. In a short time after, an old woman cured him, with a milk diet and sasafras tea.

I appeal with confidence to the members of this society, and ask them, whether they have not, in their practice, met with similar cases.

It is with the results of an extensive experience of the injurious effects of mercury in certain cases, before my eyes, that I am prepared to offer as an answer to the query, that, in some venereal diseases, mercury acts as a poison, and positively aggravates the violence of the symptoms. There is no point in the whole circle of medical opinions and practice, on which I am more completely fixed and established. I have seen it, and to my great mortification, I have had my feelings, as a professional man, lacerated by the tardy discovery of its truth. I do not know any sensation more grateful to the mind of an honest man, than the acknowledgment of error. It teaches the individual, and those with whom he is connected, that the love of truth, is superior to the love of self; and, in this view, without the slightest emotion of shame, I have no hesitation to confess, that I have, on many occasions, done much injury to my patients, by the indiscriminate use of mercury in Syphilis. But, may I not ask, as Broussais did, on an occasion when he confessed he had done wrong by not bleeding his patient, through fear of debility, "but who was to blame?" Most certainly I was not. How far my teachers, or the books they had placed in my hands were, it is not for me to decide.

The third question, "Is there any form of venereal disease that can be cured without the use of mercury?" involves principles of much importance to us all. A question recurs with great force: What, then, is meant by venereal disease?

It is not every ulceration of the genital organs. These organs are subject to a variety of ulcers, which are certainly not syphilitic, as is evident from their being destitute of the ordinary marks of chancre, the hardened edge and base; yet most practitioners look on them as such, imagining that some peculiarity of the disease's action, deprives them of their usual characters. These ulcerations, often attended by a considerable degree of inflammation, may arise from the peculiar secretions of the organs themselves, or by connection with those who are troubled by such affections. Leucorrhea in females, and particularly the discharge from incipient cancer of the uterus, will often occasion them. Mechanical abrasions, and other injuries, will sometimes cause them. No one will, for a moment, doubt the possibility of curing such ulcerations, without the use of mercury, and yet we often see that powerful remedy employed in cases of this character, where its energies are as much misapplied as they would be, in the healing of a simple incised wound.

But the views of the European non-mercurialists, go still further. Denying, totally, the truth of the axiom of Hunter, that no chancre can be healed without mercury, they say, in the language of Dr. Hennen and of Professor Thompson, "that all sores on the sexual parts may be healed without the use of mercury, in any form whatever." I am by no means prepared to go this length. I will admit no such sweeping assertion. Yet I cannot deny that there are cases of ulcers, which ought certainly to be called syphilitic, which have been cured, and which I myself have cured without mercurials.

4. Is there any reason to believe that there is more than one form of the venereal infection? Very certain it is, that the venereal disease varies much in its effects, both locally and upon the constitution. What diseases can be more different from each other in their consequences, than the true Hunterian chancre and the primary phagedenic ulcer of Carmichael, or the Black lion of the South of Europe, or Mr. Travers' Swan alley ulcer?

We want facts to prove that different individuals may not contract the various diseases, from the same infected person. All the variations of the disease may be ascribed to peculiarity of constitution; and till it can be proved that a woman, infected with any one form of the disease, shall communicate that form alone, to men who have connexion with her, the assumption of the different kinds of the syphilitic virus, is purely gratuitous. And as the varieties of constitutional peculiarities are amply sufficient to account for the varieties of the disease, according to the Newtonian principles of induction, we must wish that to rest satisfied, while the whole *onus probandi* of the question is thrown on those who espouse the opposite side.

To the 5th question, I answer, that an investigation of the history of syphilis cannot fail to produce on the mind of any one, the conviction that the disease has been, gradually, growin gmilder, and that it is not now what it once was. But at the same time, it is rather stepping beyond the bounds of truth and reason to assert, that it is not possible, at the present day, to find a real chancre on the face of the earth, and yet this opinion is held by some of the most distinguished men in this country and in Europe. My own impression is, that the disease has become milder since the days of Hunter, for, independently of the observations which I have made on the cases which have come under my particular notice, I can scarcely conceive it possible that a man of the exact and discriminating mind of Hunter, could have been so very much mistaken in the results of his numerous experiments, as to have made such a confident assertion of the truth, of what is now found to be false. But I do not stand here as the champion of that clarum et venerabile nomen. The reputation of Hunter needs no defence. How far this amelioration may enable us to dispense with the use of mercury, is not, I presume, exactly understood.

To the last question, Is mercury ever necessary? I answer yes. I believe the most violent opposers of mercury do not go so far as to exclude it entirely. Mr Hennen tells us, that in every primary sore, he would dispense with the use of mercury at first, and trust to cleanliness, rest, and abstinence.

"But," says he," if, contrary to every calculation, it remained open, I should certainly not sacrifice every dislike to mercury, knowing how many persons have been seriously benefitted by a judicious and mild administration of that remedy."

On a retrospect of what has been said, and on a general view of the whole subject, I am still strongly inclined to place implicit confidence in mercury, as the great remedy in Syphilis.

The injurious effects which this powerful agent occasionally produces, in cases of genuine syphilis, are to be attributed to some disorder or derangement of the constitution which existed before the infection.

Soon after I began to see those dreadful cases, in which the disease seems to spend its fury on the throat, bones, and periostrum, I was struck with the fact, that all these patients were of the scrofulous diathesis.

I have for many years looked at the cases which have come under my observation in this view, and I can truly say, that, with the exception of here and there a scorbutic sailor, all those instances in which lues venerea has committed its most destructive ravages, have occurred in patients decidedly strumous. The effect that this may have on the mercurial remedies, must be decided by a more extended experience, and by more numerous observations than I have been able to bring to bear on it. But this I know, that I have the utmost horror of a simple chancre on the penis of a scrofulous man; for I have generally found, that if the patient was not worn out by the suppurations of the buboes, he would sooner or later, pass through the various gradations of constitutional lues, or fall an early victim to a consumption, hurried on by this direful aggravation.

If we turn our attention, for a moment, to the men who have been most loud in their complaints against mercury, we shall find that they have acquired their experience in warm climates, where the disease is confessedly of a milder nature than with us—that they have practised in military and other hospitals, among men whose constitutions have been depraved by excesses of every kind and who were consequently very illy able to bear the use of mercurials.

The non-mercurialist allows that the constitutional symptoms are much more liable to occur in cases where the mineral has not been employed, and this of itself is a very formidable objection to their method.

In this, as in most other cases, the truth is not to be found at either extreme. Those who keep the middle path, will most probably make a nearer approximation to it than any others.

While the doctrines of syphilis are thus at loose ends, every medical man ought to be peculiarly observant of the cases which come under his care. For though we hear the words, syphilitic, syphiloidal, and pseudo syphilitic used by every one, how few of us could point out the one from the other; and how few of us can tell beforehand, whether mercury will cure or aggravate a venereal ulcer that may be presented to us.

A very ingenious writer, of late days, asserts, that no man deserves any credit for discovery in science; that these discoveries are the natural results of the gradual progress of improvements; that the next step in the march *must* be taken, and it is no credit to Franklin, or to Fulton, or to Jenner, or to Davy, that they have taken it. Be this as it may, it is very evident, that the next step in our knowledge of syphilis, must be a distinguishing of the forms of the disease, and the use of the appropriate remedies; and I do not hesitate to say, that, whether, justly or not, much praise will be awarded to him who can reduce the present chaos of professional opinions, into order and beauty.

